# Effects of computerized cognitive training for cognitive and visually impaired: one-group pre- and post-test design

**DOI:** 10.1590/1980-5764-DN-2024-0245

**Published:** 2025-09-01

**Authors:** Michael Chih Chien Kuo, Armstrong Tat San Chiu

**Affiliations:** 1 Tung Wah College, School of Medical and Health Sciences, Hong Kong SAR, China.; 2 The Hong Kong Society for the Blind, Hong Kong SAR, China.

**Keywords:** Vision Disorders, Cognitive Training, Cognitive Dysfunction, Memory, Attention, Transtornos da Visão, Treino Cognitivo, Disfunção Cognitiva, Memória, Atenção

## Abstract

Studies have shown that visual impairment is associated with an increased risk of cognitive decline. Individuals with co-existing cognitive and visual impairments are also at a higher risk of having disability, which can further impact on one’s independence in everyday activities. Computerized Cognitive Training (CCT) is one of the non-pharmacological interventions in managing cognitive decline.

**Objective:**

This study investigated the effects of an auditory CTT program in a group of participants with both cognitive and visual impairment.

**Methods:**

Sixteen participants were recruited. Participants had cognitive complaints and visual impairment. They received eight sessions of auditory-based CCT once or twice a week for 45 minutes within a 6-week period for each participant. Pre- and post-assessments were conducted before and after the training. Neuropsychological assessments included the Cantonese version of the Mini-Mental State Examination (CMMSE), the digit span forward test, the Chinese version of the Verbal Learning Test (CVVLT), and memory subtest of Neurobehavioral Cognitive Status Examination (NCSE). Pre- and post- mean assessment scores were compared.

**Results:**

Mean CMMSE score for this group of participants was 18.1. Significant differences were found in four assessments (digit span forward, CVVLT-Total: CVVLT-first trial, and NCSE-memory). No significant differences were found in the other tests. Additional repeated measures analysis showed age might play a role in CVVLT first trial and CVVLT total scores.

**Conclusion:**

The study is limited by its one-group design. The results provide preliminary evidence that auditory-based CCT may be beneficial to attention and memory in people with both cognitive and visual impairment.

## RESUMO

Estudos têm mostrado que a deficiência visual está associada a um risco aumentado de declínio cognitivo. Indivíduos com deficiências cognitivas e visuais coexistentes também apresentam maior risco de desenvolver incapacidades, o que pode impactar ainda mais a independência nas atividades do dia a dia. O Treinamento Cognitivo Computadorizado (TCC) é uma das intervenções não farmacológicas utilizadas no manejo do declínio cognitivo.

**Objetivo:**

Este estudo investigou os efeitos de um programa de TCC auditivo em um grupo de participantes com deficiência cognitiva e visual.

**Métodos:**

Dezesseis participantes com queixas cognitivas e deficiência visual foram recrutados. Eles participaram de oito sessões de TCC auditivo, realizadas uma ou duas vezes por semana, com duração de 45 minutos, ao longo de um período de seis semanas para cada participante. Avaliações pré e pós-intervenção foram realizadas antes e depois do treinamento. As avaliações neuropsicológicas incluíram a versão cantonesa do Mini Exame do Estado Mental (*Cantonese version of the Mini-Mental State Examination* — CMMSE), o teste de dígitos na ordem direta, a versão chinesa do Teste de Aprendizagem Verbal (*Chinese version of the Verbal Learning Test* — CVVLT) e o subteste de memória do Exame do Estado Cognitivo Neurocomportamental (*Neurobehavioral Cognitive Status Examination* — NCSE). As médias das pontuações das avaliações pré e pós-intervenção foram comparadas.

**Resultados:**

A pontuação média no CMMSE para este grupo de participantes foi de 18,1. Diferenças significativas foram encontradas em quatro avaliações (dígitos na ordem direta, CVVLT-total, CVVLT-primeira tentativa e NCSE-memória). Não foram encontradas diferenças significativas nos demais testes. Análises adicionais com medidas repetidas sugeriram que a idade pode influenciar os escores na primeira tentativa e no total do CVVLT.

**Conclusão:**

O estudo é limitado pelo delineamento de grupo único. Os resultados fornecem evidências preliminares de que o TCC baseado em estímulos auditivos pode ser benéfico para a atenção e a memória em pessoas com deficiência cognitiva e visual.

## INTRODUCTION

Cognitive domains, including attention, memory, executive function, and processing speed, show measurable declines with age^1^. Pathological conditions such as dementia are associated with progressive neurodegeneration and can further impact cognitive function. In addition to dementia, visual impairment is also one of the most common disorders among older populations^2^. Studies have shown that visual impairment is associated with an increased risk of developing dementia and cognitive decline^2,3^. Individuals with co-existing cognitive and visual impairments are at a higher risk of disability, which can further reduce independence in everyday activities^4^.

Management and treatment strategies for cognitive impairment include both pharmacological and non-pharmacological methods. Pharmacological interventions typically involve the use of medications designed to alleviate cognitive symptoms and correct specific neurochemical imbalances. Although these medications may provide some degree of symptomatic relief, they can lead to various side effects, and their long-term effectiveness in slowing disease progression is often limited^5^. On the other hand, non-pharmacological approaches encompass a wide range of techniques, including cognitive training, cognitive stimulation, physical activity, dietary modifications, and social engagement, all aimed at improving overall brain health, reducing risk factors, and promoting neuroplasticity^6^. For example, adopting a lifestyle that includes regular physical exercise and a balanced diet can enhance cerebral blood flow and reduce the risk of further cognitive decline^7^. Cognitive training, which usually targets specific cognitive domains, consists of structured exercises and activities intended to strengthen particular cognitive skills, thereby enhancing memory and executive function^8^. Additionally, participation in social activities can help individuals manage cognitive difficulties^9^. Non-pharmacological interventions are particularly valuable in enabling individuals to take an active role in managing their condition, potentially resulting in better long-term outcomes. Moreover, these interventions do not carry the risks and side effects associated with pharmacological treatments, making them a safer and more sustainable option.

Computerized Cognitive Training (CCT) is one of the non-pharmacological interventions used in managing cognitive decline. CCT is defined as any form of cognitive training involving the use of an individual electronic device^10^. It consists of guided drill-and-practice tasks designed to target one or more specific cognitive components. The explicit teaching of memory or problem-solving strategies is not required in the training process^11^. CCT has been increasingly applied in clinical contexts, as it is a safe, inexpensive, and beneficial method to improve cognitive functions in individuals with dementia or those at risk of developing the condition^12^. A review showed that CCT can improve cognitive domains (*e.g*., attention, processing speed, and visuospatial memory) and everyday functioning, with small to moderate effects in older adults without health conditions affecting cognition^13^. In individuals with mild to moderate dementia, a systematic review of 33 studies found that, compared to no intervention, CCT can have positive effects on global cognition and verbal fluency^14^. There is also evidence suggesting that CCT may benefit attention and memory in individuals with mild cognitive impairment^6,15^.

There is a limited number of studies investigating the effects of CCT on cognition in individuals with both cognitive and visual impairments. One apparent reason is that CCT often requires intact vision, and training materials typically involve visual stimuli and cues^10,12^. This study reported an auditory CTT protocol using commercially available software in a group of participants with both cognitive and visual impairments. Due to participants’ visual limitations, two training tasks that do not require vision and can be completed using auditory input were selected. Individual sessions and a supervised approach were employed, as these have been reported to produce greater cognitive effects^16^. The selected tasks specifically targeted selective and focused attention, and it was hypothesized that improvement in attentional performance would be observed following the training.

## METHODS

### Participants

Sixteen participants were recruited from residential facilities for visually impaired older adults. Selection criteria were as follows:

- aged 65 or above;
- reported cognitive complaints (either self-reported or reported by family members);
- visual acuity equivalent to 20/200 or worse in the better eye, indicating moderate to severe visual impairment or blindness.

Participants were excluded if they met any of the following criteria:

- inability to follow instructions;
- current use of cognitive-enhancing medication or any medication that may interfere with cognitive function;
- history of intellectual disability, central nervous system infection, brain tumor, traumatic brain injury, significant psychiatric disorders (such as major depression or schizophrenia), substance abuse, or other medical conditions (excluding dementia) that may interfere with cognition;
- communication barriers, including but not limited to deafness or other significant language impairments.

Participants were recruited through a convenience sampling method. Staff at the residential facilities reviewed medical charts of potential participants to ensure eligibility. Eight participants had a formal diagnosis of dementia. All outcome measures were conducted by occupational therapists. Ethics approval for this study was obtained from the author’s affiliated college. Written informed consent was obtained from participants or their family members prior to the start of the study.

### Assessments

Neuropsychological assessments included the Cantonese version of the Mini-Mental State Examination (CMMSE)^17^, the digit span forward test^18,19,20,21^, the Chinese version of the Verbal Learning Test (CVVLT)^22^, and the memory subtest of the Neurobehavioral Cognitive Status Examination (NCSE)^23^. CMMSE (maximum score of 30) was used to screen the overall cognitive function and was administered only at pre-test. The digit span forward test (maximum score of 9) was used to assess attention and short-term memory^19,20,21^. CVVLT, which measures working and episodic memory, consisted of nine two-character Chinese nouns with four learning trials (maximum score of 9 per trial; total maximum score of 36) and one 10-minute delayed recall trial (maximum score of 9). The memory subtest of the NCSE required participants to memorize and later recall four two-character Chinese nouns (maximum score of 12). Except for the CMMSE, all other assessments were administered within two days before and after the training sessions.

### Intervention

A total of eight individual sessions were conducted once to twice a week (every 5 to 6 days) over a 6-week period for each participant. Each session lasted approximately 45 minutes, with 30 minutes dedicated to actual training. Two cognitive training modules (CogniPlus, Schuhfried, Austria) were administered in alternating sessions. In one module, participants were required to remember and respond to targeted sounds while suppressing responses to irrelevant sounds (selective attention module; 15 difficulty levels). In the other module, participants responded to relevant auditory stimuli presented with distractions (focused attention module; 10 difficulty levels). Although the modules were designed to target attention, they likely also engaged aspects of working memory. Instructions and a practice phase were provided before the first training session. The training phase started after participants completed the practice phase. Reinforcements and reminders were offered throughout the sessions as needed. The training software automatically adjusted the difficulty levels based on participants’ performance (i.e., accuracy of responses). Each new session resumed at the level where the previous session ended. At the end of each session, participants received a performance chart summarizing recent progress. Vision was not explicitly required to complete these training modules. The training sessions were conducted by a different occupational therapist than the ones responsible for the assessments.

### Statistical analysis

Statistical analyses were conducted using the Statistical Package for the Social Science (SPSS) Statistics 26.0 (IBM, USA). Paired-samples *t*-tests were used to compare the pre- and post-assessment mean scores. A *p*-value of ≤0.05 was considered indicative of a significant difference. The Wilcoxon signed-rank test was also used to confirm the results. Additional repeated measures analyses were performed to evaluate whether trends in assessment scores differed by age range (younger 7 *vs*. older 7 years) and education level (received no education *vs*. received education). A Group (2 levels) x Time (2 levels: pre and post) model was used.

## RESULTS

Two participants did not complete the training sessions. The remaining 14 participants were all females (mean age: 85.4±11.8 years; years of education: 3.4±4.4 years). Their mean CMMSE score was 18.1±4.7 (range: 12–26). Paired-samples *t*-tests were performed to compare the pre- and post-training mean assessment scores. Results are shown in Table 1. Significant differences were found in four assessments (digit span forward test, CVVLT first trial, CVVLT total, and NCSE-memory). No significant difference was found in the CVVLT delayed recall trial. Analysis using the Wilcoxon signed-rank test showed similar results. Additional repeated measures analyses showed interaction effects between Group (age) and Time in the CVVLT first trial and CVVLT total scores (p<0.05) (Table 2); the improvements in these two tests were greater in the younger group. The two subgroups did not differ significantly in years of education (younger *vs*. older; 4.7 *vs*. 2.0) or CMMSE scores (20.8 *vs*. 15.8), and both included four participants with a dementia diagnosis.

**Table 1:** Pretest-Posttest Comparison. Data are shown as mean±standard deviation.

	Pretest (N=14)	Posttest (N=14)	*p*-value	Cohen’s d	95%CI
Digit span forward test	5.14±1.56	6.14±1.99	<0.001	-1.28	-1.45 to -0.54
CVVLT first trial	2.00±0.78	3.14±1.79	0.017	-0.73	-2.04 to -0.24
CVVLT total	11.79±4.59	13.57±6.35	0.008	-0.84	-3.14 to -0.57
CVVLT delayed recall	1.00±1.96	1.21±2.29	0.19	-0.37	-0.54 to 0.12
NCSE––memory	9.57±1.87	10.64±0.93	0.05	-0.58	-2.14 to 0

Abbreviations: CVVLT, Chinese Verbal Learning Test; NCSE, Neurobehavioral Cognitive Status Examination; 95%CI, 95% confidence interval.

**Table 2:** Additional tests for age and education groups. Results from interaction effects.

	Age x time (F value)	*p*-value	Education x time (F value)	*p*-value
Digit span forward test	2.0	0.183	0.444	0.518
CVVLT first trial	5.76	0.034*	0.109	0.747
CVVLT total	4.83	0.048*	0.928	0.355
CVVLT delayed recall	2.08	0.175	2.08	0.175
NCSE––memory	1.014	0.334	2.858	0.117

Abbreviations: CVVLT, Chinese Verbal Learning Test; NCSE, Neurobehavioral Cognitive Status Examination.

Note: *p≤0.05.

## DISCUSSION

To our knowledge, this was the first trial to include a group of aged participants with visual impairment for CCT. Results showed improvements in the digit span forward test, CVVLT first trial, CVVLT total, and NCSE-memory subtest scores. These findings provide preliminary evidence that auditory-based CCT may be beneficial for attention and memory in individuals with both cognitive and visual impairment. A previous large-scale study of auditory-based CCT in community-dwelling older adults aged ≥60 years at risk of progressive cognitive decline^24^. Their CTT aimed to improve speed and accuracy of auditory information processing lasted for one hour each day, five days a week, over a period of eight weeks. The participants in the cognitive training group showed improvements in sustained attention and working memory post training and sustained at 6 month^24^. No improvements were found in the active control or no-contact control groups. These findings support the notion of neuroplasticity, in which behavioral changes may be achieved through targeted cognitive training^25,26^, in older adults or those with similar profiles to the participants in this study. The observed improvements in this study reflect near-transfer effects (*i.e.*, in trained domains), as auditory training targeting focused and selective attention and working memory improved related areas such as digit span scores and CVVLT first trial and total scores. Far-transfer effects (*i.e.*, generalization) have been demonstrated in previous cognitive training studies^27^. However, the present study did not provide evidence for far-transfer effects, as CVVLT delayed recall scores did not significantly differ between the two time points. Additionally, results indicated that age may influence training effectiveness, with the younger group showing greater performance improvements in the CVVLT first trial and total scores.

Cavallo et al.^28^ trained a group of participants with early stages of Alzheimer disease using a computerized cognitive training program, with the aim of assessing its neuropsychological effects and determining the stability of these effects after a period of six months. Participants in the experimental group engaged with a structured rehabilitative computer program three times weekly for a duration of 12 consecutive weeks, focusing on multiple cognitive domains. In contrast, participants in the control group received a standard control intervention. Results showed that participants in the experimental group had notable enhancements across multiple neuropsychological domains. Results from Cavallo et al.^28^ and this study were similar. In addition, this study demonstrated that a relatively low intensity program (*i.e*., 8 times in 6 weeks) that focused mostly on attention and working memory could improve performance in attention and episodic memory in those with concurrent cognitive and visual impairments.

Trebbastoni et al.^29^ reported a trial that included a no-treatment control group for individuals with mild-to-moderate Alzheimer disease. Participants in the treatment group were involved in paper-and-pencil cognitive training sessions (for multiple cognitive domains) twice a week for a duration of six months, while the control group received no treatment. Results indicated those in the treatment group scored higher in general cognition, verbal fluency, naming, and episodic memory tests immediately post-intervention. Both paper-and-pencil cognitive training and CCT in this study may improve cognitive functions in cognitively impaired participants. Interestingly, even though Trebbastoni et al.^29^ trained participants on attention the digit span score did not improve after the training. Instead, the digit span performance was maintained at 6-month follow-up, while the control group deteriorated. It appeared that performance in attention could be maintained; however, whether it can be improved depends on the intensity of the training tasks.

This study reports an auditory CCT protocol using the Cogniplus software. Training tasks on selective and focused attention were selected. All participants tolerated the training sessions well and were able to complete the tasks as requested, with cues as needed. Participants showed performance improvements in attention and memory, and these improvements could be age dependent. It appeared that individual, supervised CCT approach might be beneficial, especially if the number of treatment sessions is smaller^16,28^. CCT protocols vary considerably across different studies, from a session to several sessions a week for 6 to 8 weeks or longer^8^. Training time for each session also differs between studies, ranging from 20 min to up to 90 minutes per session^8,30^. This study extended the efficacy of auditory CCT and provided preliminary evidence that a relatively low intensity protocol (one to two 45-min individual sessions per week for a total of 8 sessions in 6 weeks) could benefit cognitive capacity in those with cognitive and visual impairment^24^.

It was interesting to note that there was significant improvement on NCSE–memory but not CVVLT delayed recall test. Both tests should measure similar domains. The discrepancies may be due to differences in the administration, scoring, or complexity of these assessments. In CVVLT, the same learning list (nine two-character nouns) were read to the participants four times and the participants were tested on their memory registration after the list was read each time. On the other hand, there were no limits to the number of times NCSE–memory subtest could be read to the participants. Participants could practice unlimited times until successful repetition of all four nouns twice. Besides, if the participant could not recall a noun in the NCSE–memory subtest, categorical cues and choices were provided, and they could still get partial marks for items recalled with cues. This indicated that these participants could benefit and might need more support at the retrieval stage to show their improvements.

There were several limitations in this study. The sample size was small (especially for the sub-group analysis), and participants were more heterogenous (baseline cognitive performance differed). There was an absence of a control group, which made the results more prone to type 1 error. In addition, follow-up effects were not assessed. Nevertheless, the results of this study provided preliminary support for the efficacy of an auditory-based cognitive training program in people with both cognitive and visual impairment. In people with higher computer proficiency, the program could be adopted to allow web or mobile app-based training so that they could self-administer at home at their own time. Android or Apple devices already have accessibility functions, such as TalkBack or VoiceOver, embedded. Though the integration and adaptation would require time and careful considerations, developers such as UnoBrain has seen the need and have a goal of producing nine accessible brain games for the blind and partially sighted. Further studies that include a more homogenous sample and a control group are warranted. Investigations can also consider adding a follow-up test, *e.g*., at 1-month post-training or longer, to evaluate whether there may be long-term effects. Future studies may consider exploring the effects of multimodal programs such as CCT with physical exercises or brain stimulation and investigate whether there may be additive beneficial effects.

## Data Availability

The participants of this study did not give written consent for their data to be shared publicly, so due to the sensitive nature of the research supporting data is not available.
